# Analysing the intersection between health emergencies and abortion during Zika in Brazil, El Salvador and Colombia

**DOI:** 10.1016/j.socscimed.2021.113671

**Published:** 2021-02

**Authors:** Clare Wenham, Camila Abagaro, Amaral Arévalo, Ernestina Coast, Sonia Corrêa, Katherine Cuéllar, Tiziana Leone, Sandra Valongueiro

**Affiliations:** a London School of Economics and Political Science, Houghton Street, London, WC2A 2AE, UK; b Universidade Federal de Pernambuco-UFPE, Avenida Prof. Moraes Rego, s/n, Hospital das Clínicas, Bloco E – 4° Andar, Cidade Universitária, CEP: 50.670-901, Recife, Pernambuco, Brazil; c Centro Latinoamericano en Sexualidad y Derechos Humanos. Instituto de Medicina Social/UERJ - Rua São Francisco Xavier, 524 - 6° andar, bloco E, CEP 20550-013, Rio de Janeiro, Brazil; d Associaçao Brasileira Interdisciplinar de AIDS / Sexuality Policy Watch, Avenida Presidente Vargas 446, 13.° Floor, CEP 20071-907, Brazil; e University of Antioquia, Medellin, Colombia

**Keywords:** Zika, Abortion, Medical abortion, Global health security, Reproductive rights, Health, Emergency, Brazil, Colombia, El Salvador

## Abstract

The Zika outbreak of 2015-7 is a lens to analyse the positioning of abortion within in global health security. The sequelae of the virus almost exclusively affected newborn children, manifested through Congenital Zika Syndrome (CZS), and a focus on women at risk of, planning or being pregnant. At the global level, debate considered whether Zika would provide impetus for regulatory change for reproductive rights in Latin America, a region with some of the most restrictive abortion regulation in the world. However, regulatory change for abortion did not occur. We analyse why the Zika health emergency did not lead to any changes in abortion regulation through multi-method analysis of the intersection between Zika, health emergencies and abortion in Brazil, Colombia and El Salvador. These case study countries were purposefully selected; each had Zika infected women (albeit with differing incidence) yet represent diverse regulatory environments for abortion. Our comparative research is multi-method: framework analysis of key informant interviews (n = 49); content analysis of women's enquiries to a medical abortion telemedicine provider; and, policy analysis of (inter)national-level Zika response and abortion policies. We consider this within literature on global health security, and the prioritisation of a particular approach to epidemic control. Within this securitized landscape, despite increased public debate about abortion regulatory change, no meaningful change occurred, due to a dominant epidemiological approach to the Zika health emergency in all three countries and prominent conservative forces in government and within anti-abortion rights movements. Simultaneously, we demonstrate that regulation did not deter all women from seeking such service clandestinely.

## Highlights

• Zika affected reproductive decision making, but did not impact abortion regulation.
• Epidemiological framing ignored reproductive dimensions of emergency responses.
• Conservative forces instrumentalized disability concerns to oppose abortion rights.
• Women sought abortions clandestinely, citing Zika as a justification.
• Abortion provision must form part of health emergency planning and response.

## 1 Introduction

Health emergencies, framed as global health security threats, create distinct policy pathways. They prioritise short term outcomes to end disease transmission, rather than systemic changes which may provide more sustainable capacity to manage outbreaks by addressing the causes of the fault-lines exposed during epidemics, such as poverty, inequality and discrimination. The response to the Zika outbreak in Latin America in 2015 demonstrated this reactive approach, rooted in global health security, focusing on eradication of the vector (mosquito), development of a vaccine candidate and deployment of armed forces (Wenham and Farias, 2019).

The Zika outbreak also highlighted the impact this short-term prioritisation during health emergencies can have on women, and women's reproductive health, in a region with restrictive regulation of abortion (we use the word women to include anyone at risk of pregnancy). Congenital Zika Syndrome [CZS], manifested through microcephaly (infant born with a small or undeveloped head) and other conditions, is connected with maternal infection with Zika virus during pregnancy (Rasmussen et al., 2016). The dominant securitized policy response has been to place the responsibility onto women to prevent pregnancy (Ahmed, 2016). Such gendered policies have been criticised in contexts where up to 56% of pregnancies are unintended (Guttmacher Institute, 2016), where access to effective contraception is low, and the regulatory environment for abortion is restrictive (Hodge et al., 2016). Ninety-five percent of women in Latin America live in a country which legally restricts access to abortion. Yet, restrictive abortion laws are not associated with lower abortion rates, particularly in Latin America (Zamberlin et al., 2012; Sedgh et al., 2016), with access dependent on women's economic security (Ostrach and Cheyney, 2014), age (Shah and Åhman, 2012) ability to travel to seek termination (Jones, 2013), and if they are living in a union or married (Andersen et al., 2015).

Despite restrictive regulation, early evidence suggested that women exercised reproductive agency and continued to access abortion during the Zika epidemic, including clandestine use of medical abortion. Worldwide, medical abortion drugs are available through health systems, civil society, women's movements, pharmacies and illicit markets, although accessibility changes across locations, political spectrums and communities but also online sources (Lara et al., 2006; Távara Orozco, Chávez Alvarado, Grossman, Lara and Blandon, 2011; Zordo, 2016). Online sources may only serve a small proportion of women, but even so constitute an important proxy for analysis of women seeking abortion. An international online platform that offers medical abortion drugs (mifepristone and misoprostol) reported a statistically significant increase (up to 108% relative change between actual and expected requests (p < 0.001)) in consultations from Zika-affected countries in 2016 (Aiken et al., 2016).

In this paper we analyse how a Public Health Emergency of International Concern (PHEIC) affected abortion regulation and/or access in three Latin American countries. Given the virus predominantly manifests in new-borns as microcephaly, and prevention efforts targeted women, we sought to understand if abortion featured within country-level Zika policy decisions, and if not, why not. To understand whether different regulatory barriers prohibited women seeking abortion or led to greater integration into epidemic control protocols, we used a comparative case study of Brazil, Colombia and El Salvador. These three countries were purposefully selected; each had Zika infected women (albeit with differing incidence (PAHO, 2018)) yet represent diverse regulatory environments for abortion, with abortion legal without term limit in Colombia, illegal in Brazil, including criminalised use of medical abortion tablets, and illegal with threat of jail in El Salvador (see Table 1 for more detail on Zika incidence, predominant abortion methods, and abortion regulations). The main purpose of this paper is to understand whether abortion and reproductive rights became incorporated into the response to the Zika outbreak, and more broadly to understand what impact the health security response implemented had on women seeking abortion during the epidemic. This is important, given the current COVID-19 pandemic, where significant questions have been asked as to whether abortion is considered an essential healthcare service (Bayefsky et al., 2020). This paper demonstrates that the tension between outbreaks and abortion is not new but can act as a fore-warning for the outcomes which can occur when sexual and reproductive health (SRH) services are not mainstreamed into securitized responses to health emergencies.

**Table 1:** Zika incidence, predominant abortion methods, sexual reproductive health data and abortion regulation in Brazil, Colombia and El Salvador.

	Brazil	Colombia	El Salvador
Socio-economic indicators			
Population (World Bank, 2018)	209,469,333	49,648,685	6,420,744
Life expectancy at birth (m/f) (2018) (World Bank)	72/79	74/80	68/78
Domestic Public Health expenditure as % GDP (WHO, 2017)	9.5	7.2	7.2
GINI index (2018, World Bank)	53.9	50.4	38.6
% population with access to internet (2018, World Bank	70	64	34
Abortion Indicators			
Abortion Regulation (Center for Reproductive Rights, 2017)	Abortion permitted - In cases of rape - To save a woman's life - Anencephaly (therapeutic anticipation of delivery)	Abortion permitted - Foetal impairment - Cases of rape, incest or sexual abuse - To preserve a woman's physical or mental health	No explicit life exception: Legislation eliminated all exceptions to prohibition on abortion; availability of defence if necessity highly unlikely
Abortion Provisions	Penal Code (1940) Supreme Court decision on anencephaly (2012)	Decision C-355 (2006) of Constitutional Court of Colombia	Penal Code (1997) Chapter II, Art 133-137
Preferred Abortion methods	Misoprostol, surgical, other methods (interview with activists, percentage use unknown – no official data)	Misoprostol (50%), surgical, other chemical provision (Guttmacher, 2011)	Misoprostol, surgical (interview with activists - percentage use unknown – no official data)
Estimated # abortions (safe + unsafe)/year	1636 (Ministry of Health, 2017)/416,000 (PNA, 2016)	15,000 (Congreso de la Republica, 2018)/400,400 (Guttmacher, 2088).	20, 000 (Agrupacion Ciudadana por la despenalizacion del aborto terapeutico, 2012).
Hospitalisation linked to unsafe abortion/year (Number)	200,000 (Cardoso et al., 2020)	93,000 (Prada, Singh, Remez and Villarreal, 2011)	NA
Sexual Reproductive Health indicators			
Total Fertility Rate (births per woman) (World Bank, 2018)	1.7	1.8	2.0
% contraceptive prevalence any method (women aged 15–49) (World Bank, 2018)	80	81	72
% unmet need for contraception (% married women aged 15–49) (World Bank, 2015)	6	7	11
Adolescent fertility rate (births per 1000 women aged 15–19) (World Bank, 2018)	58	65	69
Zika Indicators			
First case of Zika	April 26, 2015	October 11, 2015	November 15, 2015
Zika cumulative cases (WHO, 2018)	231, 725 suspected 137, 288 confirmed	98,803 suspected 9927 confirmed	11, 789 suspected 51 confirmed
Zika incidence rate (susp + conf/100,000 pop) (time period) (WHO, 2018)	176.10	223.49	192.61
Zika cases in pregnant women (WHO, 2018)	26,066 suspected 11, 546 confirmed	19, 993 suspected 6, 365 confirmed	391 suspected 0 confirmed
Congenital Zika Syndrome cumulative cases (WHO, 2018)	2952	248	4

## 2 The Zika outbreak

Zika appeared in Brazil in 2015; by July 2019, 87 countries had evidence of autochthonous transmission of Zika virus (WHO, 2016; WHO, 2019). Zika transmission occurs predominantly through the vector *Aedes Aegypti*, but importantly, it can also occur through sexual transmission, heightening the need for analysing the intersection between the health emergency and SRH.

Although only 20% of those infected with Zika displayed influenza-like symptoms, and/or a rash, concern was raised about miscarriage, stillbirths and children born with CZS, manifesting with microcephaly, seizures, swallowing problems, limb contractures, epilepsy, cerebral palsy, and hearing loss (Miranda-Filho et al., 2016; Moore et al., 2017; Oliveira Melo et al., 2016; WHO 2016). It is estimated that 5–14% of babies born to mothers with a confirmed Zika infection are affected with CZS (Johansson, Mier-y-Teran-Romero, Reefhuis, Gilboa and Hills, 2016; Reynolds et al., 2017; Musso et al., 2019). The first two pregnancy trimesters are understood to be the most dangerous for teratogenic effects, yet these effects of Zika virus exposure are mostly undetected until the third trimester or birth (Brady et al., 2019). Across the Americas there have been more than 583,451 suspected cases and 223,477 confirmed cases of Zika virus infection, and 3720 cases of confirmed CZS (PAHO 2018).

### 2.1 Global health security

The policy response to Zika at national and global levels was framed within a security discourse, narrowly defined as the prevention, detection and response to emerging infectious pathogens, creating a path dependency rooted in clinical, public health and epidemiological processes to halt disease transmission at all costs, rather than a consideration of broader social determinants of health (Wenham and Farias, 2019; Ribeiro et al., 2018). Catalysed by 9/11 and anthrax attacks at the White House, the global health landscape was securitized, in an effort to ensure that highly pathogenic threats, whether human-made or naturally occurring, were re-framed as threats to the global population, alongside national economies (Davies, 2008) (Kamradt-Scott, 2015). Using a “grammar of security” (Buzan et al., 1998), the global health community began to reframe their policy response to outbreaks in this way, using language of war more traditionally associated with military threats to state survival (Enemark, 2007). The utility of this was to be able to simultaneously ensure that populations take these threats seriously, and in doing so, abide by government policy to reduce the risk of the pathogen spreading, and allow policymakers to draw on defence budgets to facilitate response efforts.

Such policy framing and rhetoric, however, evokes a exceptional response to disease. It requires the pathogen to be considered an existential threat to state survival (broadly defined) and thus permits extraordinary response efforts by government to “secure” the population from the threat, allowing governments to suspend political normality and the routine social contract that regulates the relations between states and societies (Buzan et al., 1998). The use of such an exceptionality policy frame encourages governments to focus on short term fixes to eradicate the pathogen as an enemy to the state, rather than think holistically about how the outbreak may be better managed sustainably, and nor does such an securitized response facilitate the analysis of the downstream effects of policy interventions (Wenham, 2021). This securitized approach to outbreaks has been championed globally through the International Health Regulations (2005) and the normative dialogue has cascaded down to national pandemic preparedness efforts (Davies et al., 2015), with many states now listing epidemic disease on national security strategies, hosting biosecurity teams within the military or mimicking securitized approaches in their policymaking (Michaud et al., 2019; Wenham, 2019).

The response to Zika was rooted in a security doctrine, evident by the deployment of National Health Emergency legislation in Brazil (ESPIN) and the formulation of crisis committees in El Salvador and Colombia. These were based within the Ministries of Health, and promoted an epidemiological security narrative focused on modelling the virus, vaccine development and vector control (G. d. E. Salvador, 2016; G. o. Colombia, 2016a; Government of Brazil, 2015), rather than addressing underlying socio-economic determinants which might determine a virus’ spread which include race, household ownership, urban dwelling, education, availability of water and sanitation facilities and income level (Johansen et al., 2018; MacCormack-Gelles 2018; Campos et al., 2018; Wenham and Farias, 2019).

Missing from global health security policy (broadly, and during Zika) has been the consideration of women and gender inequalities, and how such securitized policies disproportionately affect different sectors of society (Harman, 2016; Smith, 2019; Wenham, 2021). This includes the downstream effects on women's economic empowerment and labour force participation, increased feminised unpaid care work, increased domestic violence and decreased provision of SRH services. A routine part of reproductive health is abortion, yet there had been little consideration of abortion access during epidemics (Wenham et al., 2019) or other emergency settings (Lyman et al., 2018) (McGinn and Casey, 2016).

### 2.2 Abortion during health emergencies

Zika led to concern amongst pregnant women (or those planning for pregnancy), whether it would be safe to continue a pregnancy (or become pregnant) (Linde and Siqueira, 2018; Tirado et al., 2020). This concern had meaningful effects on reproductive decision making. Marteleto et al. show a 10% decline in live birth rates in Brazil between 2015 and 2016, stratified by social class (Marteleto et al., 2019) (Marteleto et al., 2020). Clearly this could have been because of multiple factors, but the suggestion is that one of those might have been women seeking to terminate a pregnancy. Yet, abortion was not explicitly mentioned in policy guidelines in Brazil, Colombia or El Salvador (Government of Brazil, 2015; Government of Colombia 2016a; Government of El Salvador, 2016). We, therefore, sought to understand if and how, in Brazil and El Salvador, despite legal restrictions, abortion was visible in policy and providers discourse; and if in Colombia, where legal grounds are wider, if it was included in the Zika policy frame, given the outbreak so fundamentally affected women's reproductive decision making. In doing so, the aim of this study was not only to further the knowledge of the impact of global health security policy on women, but to contribute to debates around access to SRH services during emergencies, and to understand how and why these were side-lined.

Neglect of abortion, whether during a health emergency or not, is symptomatic of broader conservative movements in the region and structural limitations within social and health sectors. Whether historically conservative (Colombia) or part of a regional conservative wave (Brazil, El Salvador), it was politically unacceptable for governments to countenance or facilitate discussion about abortion during the Zika outbreak (Contesse, 2019; Biroli and Caminotti, 2020). In Brazil, parliamentary conservative forces vocally rejected discussions concerning abortion during Zika. Instead, bills were proposed to guarantee additional support to women with Zika, to prevent termination. This can be understood as part of a longer trajectory starting with the Statute of the Unborn (2007) and increasing influence of the religious anti-abortion rights movement within Congress in the last decade (Correa, 2016). This political conservatism extends beyond decision-makers to those within the epistemic community of policymakers, clinicians and healthcare workers responding to Zika (Valente, 2017). In Colombia, despite legal provision for termination to support a woman's physical, mental or spiritual health (Government of Colombia, 2006), structural barriers to accessing abortion persisted, including conscientious objection, lack of health communication, cost, and logistics/distance to access services (Baum et al., 2015; Fink et al., 2016). Thus, even where access to abortion during Zika was legally permitted, women were limited in their options, and the “tyranny of the urgent” didn't appear to facilitate women's access to abortion services (Smith, 2019).

## 3 Materials and methods

Semi-structured interviews with privileged informers (those speaking in their professional capacity or representing an organisation) (n = 49) were conducted face-to-face and by teleconference in 2019 in Brazil, Colombia and El Salvador. Our sample of key informants was small and purposive. This sample size is consistent with other qualitative research on health emergencies, with at least 15 key informants in each country (Pellecchia, Crestani, Decroo, Van den Bergh and Al-Kourdi, 2015; Green J, 2013). Interviewees included: ministry of health employees, front line physicians, epidemiologists, directors of public health, abortion providers and/or facilitators (where abortion is restricted), and civil society groups to represent the range of stakeholders involved in the intersection of Zika, health emergencies and abortion. Participants were identified from a prior mapping (Wenham et al., 2019), and snowball sampling. Ethical approval was obtained from LSE [000753/08/08/18] and the Brazilian National Committee for Research Ethics (CONEP 03393618.6.0000.5208) [24/4/19]. LSE ethics approval was sufficient for research undertaken in Colombia. Given the illegality of abortion in El Salvador we did not seek ethics approval to avoid alerting authorities to research identifying criminal behaviour.

Interviews were conducted by authors in Spanish and Portuguese and were recorded, transcribed verbatim, and teams of 2 researchers reviewed each interview. Framework analysis was selected given its utility to synthesize and interpret large swathes of data, from different case studies and working across research teams with different experiences of qualitative research (Ritchie et al., 2013) (Gale et al., 2013). An analytical framework was developed and iterated through cross-referencing with literature (Wenham et al., 2019) to ensure inductive and deductive themes were included. The framework was applied to a test sample [n = 6; 2 from each case study country] of transcriptions; additional themes identified as important from the test sample were discussed and agreed by the team, added to the framework and then the framework was applied to the whole dataset.

Whilst women's experiences are pivotal to understanding the impact that Zika had on individual decision-making; we did not seek to interview women who had sought abortion during the outbreak. Instead we analysed data from 24,988 online consultations to Women Help Women [WHW] (between the emergence of Zika in Brazil 01/11/2015 to the end of the ESPIN Notification in Brazil 31/05/2017), an international activist non-profit organisation that provides information, support and medical abortion to women. For online consultation women agree “that the provided data can be anonymously analysed for statistical analysis and publication”. Because abortion decision making is often “hidden” (Nations et al., 1997), we used a methodology that included women's voices, without exposing them to reliving trauma and/or criminal risk, in order to answer the secondary question of whether policy structures and regulation impacted on women's decisions concerning abortion during Zika. Key word searches of WHW database identified anonymised requests for information, support or abortion medication. Consultations mentioning one keyword were extracted and any identifiable information removed. Common spelling mistakes were accounted for (e.g. sica, sika, zica, cica, cika). Keywords were identified through a prior analysis of 25 interview transcripts undertaken in Northeast Brazil in 2017 as part of a separate project conducted by WHW (and not our research team) to understand women's experiences with Zika and abortion (WHW & IBIS, Unpublished work, 2017). No communications were identified from Colombia and El Salvador during the same period, given different abortion regulations and informal methods for accessing contraception. In El Salvador, for example, online access to abortion medication is a lot less common than through the unlicensed “black” market and feminist groups. Content analysis were applied to the threads obtained by this search, to indirectly incorporate women's voices into our analysis. To do so we coded the justifications given for abortion in these texts into themes; Zika, socio-economic considerations, women's rights, family expectations/constraints. In each of these, we sought more nuanced themes through secondary coding across each categorisation, including stigma, relationships, financial concerns, unwanted pregnancies etc. each of which was mentioned alongside the justification of Zika for seeking abortion. Translations of quotes are authors' own.

## 4 Results

Below we present the key findings from our research, through analysis of abortion care seeking behaviours, abortion regulation, exclusion of abortion and SRH from the securitized rhetoric for Zika response, and the polarisation of abortion rights in the case study countries.

1. Abortion care-seeking

Respondents consistently agreed that women in all three countries sought abortions for reasons related to Zika: “*I have no doubt that women who had Zika or who thought they had Zika were looking to interrupt their pregnancy*” (Activist, Brazil). The WHW data demonstrated a third (32%) of requests listed Zika as the sole factor for termination. As women narrated: “*because of Zika, the foetus has not developed as it should*”; “*My child has microcephaly, they said his head is smaller than the average*”; “*I've done some tests and they detected some alterations that indicate the baby will have cranial malformation*”. These concerns echo previous studies over foetal abnormalities, and imaging to support medical decision making, contribute to decisions to terminate (Gawron et al., 2013; Horan et al., 2020). Others mentioned symptoms and assumed they had been infected “*I had a strong flu, which I don't know if it was Zika*”, or were concerned about the virus' incidence in Brazil: “*I live in Brazil and we have an outbreak of Zika virus, and there have been numerous cases of microcephaly*”. Many requests evoked language of fear in relation to Zika “*I'm very scared of the Zika virus*” and its sequelae “*Fear is taking over me…there is zika which leads to microcephaly”*.

Our interview data shed greater light in how women were seeking abortions, and the impact that a lack of regulatory change, despite the emergency had on their trajectories: regulation, and the inability of the global health landscape to open up to debates concerning SRH. “*In my country [Brazil] abortion is considered crime, even with the outbreak of Zika virus*”; “*Because abortion is forbidden, I am having difficulties finding the medicine*”, and more tangible risks that they felt: “*if I do ask for medicine, will they be stuck at the customs?*”, or raised concern of what might happen to them: “*I tried to buy misoprostol in my town, but have learned that people went to jail because of that*”.

2. Abortion regulation

Our key informant interviews discussed the impacts of this abortion regulation. One narrated that a Mexican colleague joked that “*he had been learning Portuguese*" because many of the patients at his abortion clinic in Mexico City (where abortion is legal) were from Brazil (Healthcare provider, Brazil). This was mirrored in El Salvador where participants suggested those who could afford it “*would also go to Mexico City for this service*” (Healthcare provider, El Salvador). Yet, importantly, this would likely only have reflected a small proportion of women seeking terminations who would be able to afford such an option for international travel, increasing concerns of stratified reproduction (Colen, 1995) (Johnson, 2017).

Private healthcare practitioners in Brazil and El Salvador stated that they had seen women seeking termination, both legally, such as pregnancies where CZS put the woman's life at risk, permitting medical termination of pregnancy (under contemporary Brazilian regulation) and illegally, fearing for their pregnancy in the time of Zika (Policymaker, El Salvador; Healthcare provider, El Salvador; Healthcare provider, Brazil). Respondents noted that it was an open secret that there was an increase in the number of medical abortions in Brazil although not formally recorded due to illegality (Healthcare provider, Brazil). Respondents in El Salvador noted there had been several locations where people were able to procure Misoprostol during the outbreak (Healthcare provider, El Salvador).

These experiences reflected cases outside of the Brazilian and Salvadorian public health systems. Respondents from within the Brazilian public health system (SUS) did not report similar requests for abortion: “*I didn't see anyone who talked of abortion…. I didn't have anyone come for a consultation [raising the topic of abortion]*” (Healthcare provider, Brazil). However, issues of abortion and Zika likely never appeared within the SUS because the SUS rarely had access to the serology reports [to confirm Zika infection] (Healthcare provider, Brazil) and because Zika was not detected until late in a pregnancy, women may not have thought abortion would be an option, even clandestinely (Healthcare provider, Brazil). Moreover, it might reflect the lack of willingness of public sector employees to discuss abortion, because of their own views or because they are afraid of the conservative movement in society and within the public health landscape itself.

As abortion is legal in Colombia, this permitted women to seek abortion should they wish. One provider noted an increase in abortions in 2016 (14,000 abortions) compared to 2015 (10,000 abortions), however clinical records do not account the reason(s) for termination. Although the increase in abortion rates cannot be consistently attributed to Zika, we can hypothesize that a correlation exists. (Service Provider, Colombia).

3. Exclusion of SRH from health security rhetoric

Despite incomplete evidence suggesting the use of abortion in response to Zika, the narrowness of the global health security rhetoric did not facilitate discussion for policy change or systematic national dialogue about abortion in these three countries. Instead, we saw the continuation of the securitized approach to Zika, focused on the epidemiology and clinical medicine.

This approach to policymaking focused on minimising the transmission of the disease: *“From the start, everything focused on epidemiological control and the illnesses”*(International Organization, El Salvador)*; “all the efforts involved preventing the vector, but there was nothing on the theme of sexual and reproductive rights”* (Healthcare provider, El Salvador). Whilst there was no question that epidemiology and public health officials were vital, these narrow disease control efforts excluded health promotion efforts or explicit consideration of women. Emergency committees created failed to include the Ministries of Women or any SRH constituencies within their membership (Government of El Salvador, 2005; Government of Colombia, 2016b): “*Why didn't someone from reproductive health sit on the crisis committee?... this shows the disarticulation, lack of harmonization, disconnection, between the [epidemiology and SRH] processes*” (Service Provider, Colombia). Similarly, in Brazil it was noted “*What we had to do was to fight a mosquito, and that was that. Nothing new [for SRH] was going to be put in place*.” (Activist, Brazil). This was mirrored in El Salvador. Reflecting socially conservative values prevailing in the case study countries, one suggestion for this lacuna in expertise was that it was less socially difficult for policymakers to bury their head in the sand about reproductive rights: *“The Ministry of Health… can't say wear a condom, that happens in a doctor's clinic… it is much easier for them to say “we're coming to fumigate”* (Activist, El Salvador).

4. Polarisation of abortion rights debates/support

Whilst the response to the outbreak was being managed almost exclusively from the epidemiological perspective, in a parallel path, an increasing polarisation around abortion rights was registered at multiple levels of governance. As the vice minister of health in El Salvador narrated: *“Since 2016...the debate about the decriminalization of abortion had grown in strength…. But these were parallel themes [to that of the Zika response] that had no relation to the broader needs of the population… and were promoted by social groups and women's groups”* (Policymaker, El Salvador).

4. a Right to decide

Right to decide advocacy had increased in El Salvador since the case of Beatriz in 2013 (Bougher, 2017), unifying feminist civil society to push reproductive rights and the depenalisation of abortion (Policymaker, El Salvador). This was mirrored in Colombia, where despite regulatory change in 2006, “*discussions of reproductive health remained amongst the women's movement*” (Service Provider, Colombia). In Brazil “*there was a lot of pressure from women's movements to raise the need for guaranteed access to safe abortion*” (Healthcare worker A, Brazil). Thus, for many there was a perception that any Zika-abortion debate occurred almost exclusively within women's movements. One respondent identified this within a broader trans-national feminist action: “*Abortion I see this as from a movement of women from abroad applying this to the context of Zika*” (Healthcare provider, Brazil). As one participant stated: “*for many health professionals, this debate appeared as an articulation of crazy feminists…*” (Healthcare provider, Brazil) rather than meaningful engagement with safe abortion as part of universal healthcare or a response to a health emergency. Yet, it appeared from the medical professionals and service providers that we interviewed that this debate was occurring on the micro level in consultations across case study sites and articulated as individual options rather than debates on regulatory change.

A notable example of this feminist movement was Debora Diniz and Anis-Instituto de Bioetica who led the Arguição de Descumprimento de Preceito Fundamenta (ADPP 5581/2016)) a petition tabled at the Supreme Court of Brazil (Correa, 2018). The petition sought rights for women and those at risk of infection from Zika, including access to: Benefício de Prestação Continuada (Continuous Cash Benefit Programme); therapy services for children born with CZS; clear risk communication about the virus to the Brazilian population; and, SRH services (including abortion) (Anis, 2016). Respondents identified this as a ground-breaking moment in Brazil, with active judicial strategic litigation to alter abortion regulation. This first petition was also important because it paved the way for the tabling, in March 2017, of ADPF 4442/2017, a new lawsuit requesting the complete decriminalization of abortion up to 12 weeks: “*Together these had a huge effect on media coverage and public discourse, both in terms of news coverage concerning abortion, but in terms of quality and which experts were listened to*” (Activist, Brazil). In May 2020, the Supreme Court rejected ADPF 5581/2016 on technical grounds but within a political climate where the Bolsonaro administration was already openly aligned with the anti-abortion rights movement (Saldana, 2020).

Notably missing from the right to decide conversations were government institutions (Healthcare provider, Brazil), and for many this remained the limiting factor – the debate was generated by the feminist movements, and remained there, distinct from the health security approach of the national governments (Activist, Brazil). When the Ministry of Health officials came to discuss the outbreak, “*this was always focused on epidemiological data…. And a discussion of women was outside of this discourse"* (Healthcare provider, Brazil D). This contrasted to the debates of SRH access. In Pernambuco, one respondent representing the mothers of children born with CZS suggested that the state government's slow response to consider SRH rights was because government activity is just slow and bureaucratic (Activist, Brazil), yet others feared this was strategic, and that this omission actively demonstrated what was considered part of the securitized response and what was not (Healthcare Provider, Brazil; Activist, El Salvador).

Instead of engaging with abortion debates, each government precluded such discussions through recommendations that women should avoid or delay pregnancy during the peak of the epidemic (Government of Colombia. Ministerio de Salud, 2016). Several of our respondents discussed the role this had in silencing broader discussions around SRH and/or abortion (Activist, Brazil; Activist, El Salvador; Service Provider, Colombia), which was particularly dangerous given that the disease also spreads sexually (Healthcare Provider, El Salvador). In doing so, this *“once again responsibilised women”* (Activist, Brazil). Such recommendations fail to take into consideration women's rights, structural barriers which prevent women from becoming pregnant and raises broader questions around access to SRH services during outbreaks (Anonymous, forthcoming; Diniz, 2017; Anderson 2015).

In El Salvador, Zika provided a potential policy window for SRH, yet, the two parallel processes of epidemiology and abortion connected in very few institutional and social spaces (Healthcare provider, El Salvador). Those civil society organisations that promoted the decriminalization of abortion continued with broader messages of decriminalization (on any grounds, not just on account of Zika) rather than denoting Zika as a justification for which abortion was allowed (Activist, El Salvador; Policymaker, El Salvador). Moreover, the Ministry of Health did not incorporate abortion as a clinical trajectory in its Zika discussions and planning (Policymaker, El Salvador). Thus, whilst Zika became an element in the broader national debate on the decriminalization of abortion, the health emergency did not materialise into any institutional change. As one activist organization noted, this might have been on account of the relatively low numbers of CZS and microcephaly in El Salvador in comparison to Brazil, and thus it didn't pose a major problem clinically and/or as a social debate (Activist, El Salvador).

In Colombia “*Zika was an opportunity to reposition the debate about abortion as an integral health topic*” (Activist, Colombia). Yet, despite abortion being legally permissible on the grounds of a mothers' physical or mental health (which could include Zika), “*there was no discussion of abortion*” (Healthcare provider, Colombia) within Zika policy decision making in Colombia and it remained an epidemiological concern, rather than a gendered policy issue (Service Provider, Colombia). Some respondents lamented this missed opportunity “*it could have been used as a moment to leverage … the social decriminalization of abortion, because it may be legally decriminalized but still in the minds and imaginations of Colombians that is not has happened” (*Service Provider, Colombia*)* and a lack of explicit consideration of Zika in policy documents didn't provide broader change to the abortion landscape*.* Despite progressive abortion laws, the Zika outbreak demonstrated that abortion remains *“a taboo at the social level and political level, even with political representatives who are pro-abortion, they find it difficult to discuss openly because of the social conservative society”* (Activist, Colombia).

This social conservativism is further challenged by structural barriers to accessing abortion services, including: low levels of awareness of the legality of abortion by many physicians and the general population; stigma and discrimination against abortion providers; inadequate or absent training of abortion providers (Service Provider, Colombia). The result of which was that many women – particularly poor, rural, indigenous, migrants - remained without access to legal, safe abortion during Zika (Activist, Colombia) (Policymaker, Colombia). Several respondents noted that these issues could have been reduced had abortion been systematically featured within Zika debates and policymaking. But the government position was that it was a legal option to all women, and as such it didn't need to be expressly included in Zika policy.

Moreover Colombia's broad legal provision for abortion during Zika highlighted a secondary problematic issue - the difficulties in accessing later abortion ( Government of Colombia, 2006). The Constitutional Court had not put a gestational limit on abortion; stating women had the same rights at any stage of their pregnancy given that their physical or mental health might be affected in any trimester (Policymaker, Colombia). CZS is usually identified in the second or even third trimester and thus, several women sought later abortions in Colombia on account of Zika (Activist, Colombia; Policymaker, Colombia).

This caused logistical challenges; several providers only had facilities to terminate pregnancy up to 24 weeks (Policymaker, Colombia), and outside of major cities (notably those above the altitude for mosquito bites) there was no provision at all (Policymaker, Colombia). Accordingly, service providers in rural locations subsequently had to develop capacity to deliver late stage terminations (Policymaker, Colombia). Elsewhere, women's organisations accompanied women to facilities in cities to perform abortions at later gestational age (Policymaker, Colombia). This added a new element to SRH provision in Colombia, but one which was not actively highlighted or promoted by feminist groups, for fear of protestors.

4. b Anti-abortion rights pressures

In all three countries, anti-abortion rights ideologies, permeated government structures and was internalised within the securitized response to Zika. This anti-abortion sentiment came from both the top down and bottom up, squeezing opportunities for open debate and regulatory change.

At the macro level, increasingly dominant conservative forces in congress in Brazil and El Salvador during the outbreak collaborated with the religious, anti-abortion movements to actively resist efforts to (re-)open national debates concerning Zika and abortion, fearing “*the mosquito was an ally for the depenalisation of abortion*” (Activist, Brazil; Activist, El Salvador). These voices of the anti-abortion rights movement also dominated the epistemic communities of clinicians and scientists (De Assis Machado & Maciel D, 2017; Gressick et al., 2019). Yet it is unclear whether the power these voices had amongst policymakers was the result of active decision making to support this position, or the result of the omission of SRH concerns which fell outside of the securitized response.

At the micro level, in Brazil, women with children born with CZS were perceived as being instrumentalised by anti-abortion rights groups to become vocal narrating “*You have a child like this because God gave you this opportunity to grow more, to learn more with this suffering*” (Healthcare worker, Brazil). Discourse about these cases referred to these mothers as “*martyrs, courageous, warriors*” for not choosing termination (Healthcare provider, Brazil). In one instance, mothers of children with CZS were encouraged by anti-abortion organisations to participate in a forum with women in Pernambuco concerned about Zika to get women to reconsider abortion they might be considering (Healthcare worker B, Brazil). Women from the mother's associations of babies born with CZS tended to be against abortion *“I'm against abortion… and I represent many here… who are also against this”* (Activist, Brazil), reflecting the range of perspectives during Zika within our case study countries and cautioning generalisations.

In El Salvador, the anti-abortion movement continued to assert its position publicly, and with decision makers who controlled the legislative assembly, although the limited prevalence of babies born with CZS meant that this was no more vocal than in non-epidemic times (Policymaker, El Salvador). However, the government made it clear that they would not open a political debate. As one respondent *suggested “The recommendation was not to get pregnant, nothing more”* (Activist, El Salvador). Described by one respondent as *“here it's like under the Taliban, women do not have rights to decide anything… and if women do appear in emergency rooms [having sought termination] we see them handcuffed”* (Healthcare provider, El Salvador). Indeed, in broader discussion of abortion with Ministry of Health officials we were told *“we don't have maternal mortality from septic abortion, we don't have septic abortions, so we don't have deaths from unsafe abortion, so to speak”.* Such silencing had ramifications as to how medical professionals were un/able to advise and discuss abortion with women during the outbreak. As one stated: “*This was not right and [the government] could have pursued another plan, another context that was a little more embedded in the national reality”* (Healthcare provider, El Salvador).

In Brazil, in particular, during the Zika crisis, the anti-abortion rights movement established links with the disability rights movement. This meant that public debate concerning Zika as a ground for abortion was positioned against disability and diversity in society, and the human rights of those who live with disabilities (Healthcare provider, Brazil). As one respondent suggested: “*I believe very much that the strategy… to question eugenics was a global strategy, even financed globally… that abortion should not be the on the agenda of a health emergency”* (Activist, Brazil)*.* In El Salvador, as well, one concern was that if Zika became an exception for abortion debates, that might have ramifications for pregnant women carrying children with other conditions. As one respondent stated: “*How do I treat a child with Down's Syndrome? How do I treat a child with paralysis?”* (Activist, El Salvador).

In Brazil, the anti-abortion connection with the disability rights movement has gained further political cloud after the 2018 election, as the new administration has strong links with disability rights groups and this has raised the topic to the highest policy level (Policymaker, Brazil). This implied the further marginalization of reproductive rights and abortion movements by the state. As one activist stated “*This was very smart of them [the anti-abortion rights movement] as it allowed them to use secularized language around the rejection of abortion… so it is not about protecting life, the holy life from a Christian perspective. But the thing is that they are protecting children with disabilities, and they are protecting their human rights, and they're protecting their dignity, and they're protecting health”* (Activist, Brazil). Thus “*anyone who aborted [on account of Zika] would be making an eugenic decision*” (Healthcare Provider, Brazil). Even within Brazilian feminist movements, disability rights concerns raised internal disagreements over concerns of eugenics so that some prominent feminists came out against abortion during Zika (Activist, Brazil). Many, however, disagreed, arguing that this was not eugenics, as *“eugenics was a state policy to eliminate people who were not perfect”* and as the state was not opening up regulatory change to permit abortion, then this could not be eugenics (Healthcare Provider, Brazil).

## 5 Limits of study

We sought to ensure representation amongst our interview participants, inclusive of policymakers, healthcare providers, service providers and activists, but we recognise these views do not reflect all experiences and perspectives on Zika, abortion and health emergencies in Brazil, Colombia and El Salvador. We have sought to overcome this through triangulation of the literature and research methods (Mayring, 2004). This is particularly important in the case of Brazil where we only had ethical approval for research in Paraiba and Pernambuco, the epicentres of the Zika outbreak, and may not represent the diverse Brazilian landscape socially or in relation to the abortion debate. The qualitative data presented references interviews conducted with individuals in their professional capacity. We have not included details of their employers or names in line with ethical process, but we have identified the sector they represent. We recognise that these individuals come from different sectors with different interests, epistemic and moral norms. We wanted to give each of these the same weight in our analysis and reduce our own bias, but we recognise that this comes with its own challenges relating to the marshalling of evidence between a woman's personal decision, and a policymaker's professional role.

We also recognise that the sample used from WHW requests is self-selecting, and only refers to requests from Brazil. Our analyses of online consultation data, however, represents a novel source of evidence from women without exposing them to the potential risks and harm of participating in research (Larrea et al., 2015). These perspectives exclude those who were unable to, did not want, or sought abortion from another source. We sought to include further anti-abortion rights perspectives in our key informant interviews; our requests for participation went unanswered. Finally, we undertook the field research for this study in 2019, three years after the start of the Zika outbreak. Although Zika is still circulating in each country, it no longer remains a policy priority and asking participants to reflect on what happened three years ago may mean that key discussions are forgotten and/or selective memory has occurred (Stiles, 1993).

## 6 Discursive conclusion

Zika provides a critical lens to analyse whether a health emergency response embedded within norms of global health security affects policy or practice of abortion. For a health emergency which predominantly manifests in neonatal malformation, reproductive rights and access to safe abortion whilst they became part of the debate, did not feature anywhere meaningful in policymaking. We suggest this is because of two factors. Firstly, the narrow policy response using internalised understandings of global health security which focus on the epidemiology and fail to consider the downstream effects on women and access to SRH services. Secondly, deeply held normative institutional and cultural beliefs have reduced, and even eliminated, broader debates concerning access to abortion. Accordingly, we find a demonstrable difference of focus and prioritisation between those creating policy and those (women and women's groups) affected by the Zika response and SRH regulations (Seckinelgin, 2017).

Whilst Zika reinvigorated discussions of reproductive rights at multiple levels of analysis amongst transnational feminist movements, the securitized pathway limited impact or debate on regulatory change for abortion. Indeed, if anything Zika intensified pre-existing strongly held positions. As one respondent put it “*Zika opened the forum to talk [about abortion] but it stayed just at that – talking”* (Healthcare worker, El Salvador) and governments continued to side-line any formal debate concerning abortion during the Zika crisis, and the status-quo remained. Government silence on abortion provision during Zika was, in effect, tacit agreement with the conservative anti-abortion rights movement.

Given that regulation of abortion is governed at a national level, regulated by executive and legislative branches of government, our analyses demonstrate a disconnect between current policy and what women are doing in practice. Even where regulation doesn't permit termination, we know that fertility dropped (Marteleto et al., 2019), and our data validate that some women sought clandestine abortions on account of Zika, or their perceived risk of CZS, and many were unable to do so safely. Official statistics either not exist (eg: El Salvador), or where they do exist (eg: Colombia) are rendered useless because they combine spontaneous and induced abortions, with no possibility of disaggregation.

When women did seek termination due to concerns about Zika, they were limited by regulatory structures criminalising abortion and/or barriers to accessing safe abortion (DePiñeres et al., 2017). Women's access to abortion in legal settings is heavily dependent on healthcare provider awareness and willingness to offer guidance (Aniteye and Mayhew, 2013; Harries et al., 2009; Ramos et al., 2014). Ultimately, women who wanted to terminate a pregnancy as a consequence of Zika may not have been able to (Marteleto et al., 2017). We suggest that legal and structural barriers to accessing abortion are amplified by a health crisis where heightened fear and increased anxiety may magnify such tensions (Kinsman, 2012; Yang et al., 2018), and thus the global health security regime must find a way to incorporate SRH provision into policies developed to minimise disease transmission.

We argue that the failure for a meaningful national discussion on reproductive rights as part of the response to the Zika outbreak was the dominance of global health security's biomedical, clinical, public health and epidemiological narratives (Kelly et al., 2020; Harris et al., 2016) in Brazil, Colombia and El Salvador. Whether this was strategic decision or a downstream effect of the dominance of global health security narratives within mainstream response to national disease control, the result was that SRH was ignored (González Vélez and Diniz, 2016; Roa, 2016). As one respondent summed up; “*the focus was in the hospitals and there – nobody understood women's rights or even reproductive rights, we didn't understand the significance of such rights*” (Quasi-Government Official, El Salvador). This precluded considerations of a rights-based approach to the response, to consider reproductive rights, or broader socio-economic determinants of infection (Rasanathan et al., 2017). Women were left without autonomy over their reproductive health and ultimately bore the brunt of the outbreak (Davies and Bennett, 2016; Wenham, 2021). Women were made responsible for (not) becoming pregnant and placing their (unborn) child at risk of infection (Wenham, 2021), unable to continue paid employment due to the care demands of a child with complex health needs (Human Rights Watch, 2017; Bardosh, 2019). These outcomes were amplified as many partners of women with children of CZS abandoned them before or after birth (Human Rights Watch 2017). Thus, the absence of gender-mainstreaming (an approach to policy making which ensures gender equality and sensitivities incorporated into policies) and/or considerations of reproductive rights within the national responses to Zika is notable. This reflects broader trends in global health policymaking whereby a lack of representation of women and gender expertise can lead to significant gaps in policy development (Braidotti et al., 1994; Devlin and Elgie, 2008; Swiss et al., 2012). The failure to engage women's groups and representation within the securitized response and planning for Zika meant that changes to regulation for abortion and/or sexual and reproductive health would ultimately be limited.

Our comparative evidence underscores an urgent need to better link sexual and reproductive health, including abortion, in global health security. Whilst SRH and humanitarian emergency linkages are relatively well-established (Palmer and Storeng, 2016), health emergencies are rarely considered, and we argue as a result of the narrow policy focus created to respond to disease control. This need is particularly urgent given COVID-19, where self-isolation and/or mandatory quarantine limit access to sexual reproductive health services if people are unable to reach services (Schaaf et al., 2020; Todd-Gher and Shah, 2020), and/or people may fear seeking such services if they consider clinics to be a source of infection and/or such services may be reduced or cease if they are considered to be non-essential (Hussein, 2020). Understanding the impact of a health emergency on abortion decision making is vital to reduce abortion-related morbidity and mortality and ensure that women's needs are recognised and provided for within epidemics.

## Credit author statement

CW, EC, SC, TL conceived and designed the original research project, from which this paper developed. Further design of the project was undertaken by all authors after grant award. CA, AA, KC, SV and CW undertook data collection, CA, AA, SC, KC, SV and CW undertook data analysis and framework development. EC and TL contributed to conceptualisation. CW drafted the paper, with input from all authors. All authors reviewed, edited and approved the final version.
